# Novel *MKRN3* gene mutation associated with central precocious puberty in a Chinese child: a case report

**DOI:** 10.3389/fendo.2024.1491664

**Published:** 2024-12-20

**Authors:** Jingna Wang, Rongmin Li, Jieying Wang, Di Wu, Shuqin Lei, Yanmei Sang, Jie Chang

**Affiliations:** ^1^ Baoding Hospital, Beijing Children’s Hospital Affiliated with Capital Medical University, Baoding, China; ^2^ Key Discipline of Pediatrics, Endocrinology Genetics and Metabolism Center, National Center for Children’s Health (Beijing), Beijing Children’s Hospital Affiliated with Capital Medical University, Beijing, China

**Keywords:** idiopathic central precocious puberty, MKRN3, Chinese child, c.1219delA, p.R407Gfs*75

## Abstract

**Objective:**

The objective of this study is to investigate the clinical presentation and underlying genetic etiology of a Chinese child diagnosed with idiopathic central precocious puberty (ICPP).

**Methods:**

Clinical data from a pediatric patient with ICPP, including medical history, physical examination findings, laboratory results, and imaging studies, were collected and analyzed. Whole exome sequencing (WES) was performed to identify potential pathogenic genetic variants underlying the patient’s ICPP.

**Results:**

A 4 ¾-year-old female patient presented with precocious puberty, characterized by accelerated growth, Tanner stage II breast development, and Tanner stage I pubic hair. A café-au-lait macule was observed on the patient’s right flank. WES revealed a novel makorin RING finger protein 3 (*MKRN3*) gene heterozygous frameshift pathogenic variant c.1219delA (p.R407Gfs*75), which was inherited from the patient’s asymptomatic father, and leading to a truncated protein 73 amino acids downstream from the mutation site.

**Conclusion:**

This case underscores the genetic heterogeneity of ICPP and further implicates *MKRN3* gene mutations in its pathogenesis. The identification of this novel pathogenic variant expands the known mutational spectrum associated with ICPP, particularly within the Chinese pediatric population. Comprehensive genetic testing should be considered in pediatric patients presenting with early-onset ICPP to facilitate accurate diagnosis, inform genetic counseling, and guide personalized management strategies.

## Introduction

Central precocious puberty (CPP) is a common pediatric endocrine disorder characterized by the premature activation of the hypothalamic-pituitary-gonadal (HPG) axis, resulting in the development of secondary sexual characteristics before the age of 8 years in girls and 9 years in boys. This pediatric endocrine disorder exhibits a notable gender disparity in its prevalence, with a significantly higher incidence in girls. Epidemiological data suggest that the prevalence ranges from 0.217 to 26.28 per 10,000 in girls, compared to a much lower range of 0.02 to 0.9 per 10,000 in boys (1). This gender disparity suggests that genetic factors may play a critical role in the pathogenesis of CPP. Although the precise molecular mechanisms remain under investigation, genetic contributions are emerging as a key area of research in understanding this condition. Previous studies have identified the makorin ring-finger protein 3 (*MKRN3*) gene as a crucial factor in the onset of puberty. MKRN3 negatively regulates the HPG axis, and loss-of-function (LOF) mutations in this gene can lead to increased gonadotropin-releasing hormone (GnRH) secretion, premature activation of the HPG axis, and subsequent development of CPP (2–4).

The *MKRN3* gene, which encodes a RING (really interesting new gene) zinc finger protein, was first identified in 1999 by Jong MT et al. during their investigation into the molecular mechanisms of Prader-Willi syndrome (5). Located on chromosome 15q11.2, a critical region associated with Prader-Willi syndrome, *MKRN3* is a single exon, paternally expressed imprinted gene that encodes an E3 ubiquitin ligase. MKRN3 protein contains multiple domains, including two C3H motifs at the N-terminus, followed by a unique makorin-type Cys-His (CH)-rich domain, a C3H4 RING zinc finger, and a terminal C3H motif. The RING zinc finger domain is responsible for its E3 ubiquitin ligase activity, while the C3H zinc finger motifs are involved in RNA binding. MKRN3 plays a role in protein degradation and is involved in various cellular processes, including signal transduction, cell cycle regulation, differentiation, and morphogenesis (6).

While numerous studies have explored the genetic underpinnings of CPP, data from the Chinese population remains limited. This report presents a case of CPP in a Chinese child attributed to a novel *MKRN3* gene mutation, aiming to enhance clinical understanding of this condition and its genetic determinants.

## Clinical presentation and genetic analysis

### General information

A 4-year-9-month-old female patient presented to our hospital on March 17, 2021, with a chief complaint of “breast development for 2 months”. At birth, a congenital melanocytic nevus measuring approximately 1.5 cm × 2 cm with irregular borders was noted on her right waist. At 4 years and 7 months of age, the patient experienced thelarche, which spontaneously regressed without medical intervention. However, two weeks prior to this admission, the patient again noticed recurrent breast development.

### Personal history

The patient is the firstborn from an uncomplicated, full-term cesarean section delivery, with no history of perinatal complications. Birth weight was 3.4 kg, and birth length was 50 cm. Developmental milestones, including gross motor skills and social interaction, were appropriate for age. The maternal prenatal course was unremarkable. There is no history of consanguinity between the parents. The father, with a height of 167 cm, experienced his pubertal growth spurt at approximately 12 years of age, with growth cessation at 14 years. The mother, with a height of 162 cm, experienced menarche at 13 years of age.

### Physical examination

The patient’s height was 116 cm, which is slightly above the average for her age (109 ± 4.1 cm), with a weight of 20 kg and a BMI of 14.9, all in the normal range (17.8 ± 2.4 kg and 15.3 ± 1.6). No dysmorphic facial features were observed. A 3 cm × 4 cm irregular café-au-lait macule was present on the right waist. There was no evidence of acne or dorsocervical fat pad. The thyroid gland was non-palpable. Breast development was consistent with Tanner stage II, with bilateral breast buds measuring approximately 1.5 cm × 1.5 cm, and no areolar hyperpigmentation. The external genitalia were age-appropriate, with the labia majora fully covering the labia minora. Pubic hair development was consistent with Tanner stage I, and no axillary hair was present. The spine and limbs showed no deformities, and the neurological examination was unremarkable.

### Laboratory investigations

Laboratory tests revealed a normal complete blood count, comprehensive metabolic panel, and urinalysis. Tumor markers, including alpha-fetoprotein (AFP), carcinoembryonic antigen (CEA), and carbohydrate antigen 19-9 (CA 19-9), were within normal limits. Thyroid function tests, including thyroid-stimulating hormone (TSH) at 1.75uIU/mL (reference range:0.4-6uIU/mL), free thyroxine (free T4) at 17.24pmol/L (reference range:8.37-29.6pmol/L), free triiodothyronine (free T3) at 6.96pmol/L (reference range:2.5-9 pmol/L) were all within normal ranges. Insulin-like growth factor 1 (IGF-1) at 262 ng/mL (reference range: 49-283ng/mL) was normal. Adrenocorticotropic hormone (ACTH) was measured at 13.92 pg/mL (reference range: 0 - 46 pg/mL), and cortisol was within the normal range at 273.30 nmol/L (reference range: 138.2 - 691 nmol/L).

Sex hormone analysis showed the following levels: luteinizing hormone (LH) at 0.3 IU/L (reference range: 0 - 0.33 IU/L), follicle-stimulating hormone (FSH) at 2.14 IU/L (reference range: 0.42 - 5.45 IU/L), prolactin at 7.64 ng/mL (reference range: 4.2 - 23.04 ng/mL), estradiol at 15.08 pg/mL (reference range: 0 - 10 pg/mL), total testosterone at 12.64 ng/dL (reference range: 1.1 - 62 ng/dL), and progesterone at 0.21 ng/mL (reference range: 0 - 0.35 ng/mL), as shown in **Table 1**.

**Table 1:** The main laboratory data of the patient.

Laboratory tests	result	reference range
TSH (IU/mL)	1.75	0.4-6
FT4 (pmol/L)	17.24	8.37-29.6
FT3 (pmol/L)	6.96	2.5-9
IGF-1 (ng/mL)	262	49-283
ACTH (pg/mL)	13.92	0 - 46
Cortisol (nmol/L)	273.3	138.2 - 691
LH (IU/L)	0.3	0 - 0.33
FSH (IU/L)	2.14	0.42 - 5.45
Prolactin (ng/mL)	7.64	4.2 - 23.04
Estradiol (pg/mL)	15.08↑	0 - 10
Total testosterone (ng/dL)	12.64	1.1 - 62
Progesterone (ng/mL)	0.21	0 - 0.35

The arrow (↑) indicates that estradiol levels are higher than the normal range, which may be associated with precocious puberty in the child. However, estradiol levels can vary significantly and should not be used as a diagnostic criterion for CPP.

### Imaging studies

Breast ultrasound revealed bilateral breast buds with the left breast measuring 2.9 cm × 0.7 cm × 3.4 cm and the right breast measuring 2.9 cm × 0.6 cm × 2.9 cm. The margins were ill-defined, with no significant internal abnormal echogenicity or increased vascularity detected.

Pelvic ultrasound showed a uterus measuring 3.0 cm × 1.5 cm × 0.9 cm with a thin endometrial stripe. The left ovary measured 2.4 cm × 0.7 cm × 1.0 cm, containing two follicles each measuring 0.3 cm, while the right ovary measured 2.9 cm × 1.0 cm × 1.2 cm, containing six follicles each measuring 0.3 cm.

Abdominal ultrasound showed no evidence of adrenal masses or enlargement. Brain MRI demonstrated a normal-appearing pituitary gland.

### Genetic analysis

All clinical investigations and therapeutic interventions were performed with informed consent from the patient’s legal guardians and were approved by the institutional ethics committee.

After obtaining informed consents, 3 mL of EDTA-anticoagulated peripheral blood was collected from the patient and her parents. High-throughput whole exome sequencing (WES) was performed by Beijing Maikeno Technology Co., Ltd.

Briefly, genomic DNA was extracted from the blood samples and subjected to DNA library preparation. The library was then hybridized with biotinylated probes targeting the whole exome (P039-Exom, Maikeno) under optimized conditions. Streptavidin-coated magnetic beads were utilized to capture the biotinylated probe-target DNA complexes. The captured DNA fragments were then eluted, purified, and enriched. The enriched target DNA was sequenced using the Illumina NextSeq 500 platform. Sequencing reads were aligned to the human reference genome hg19 using the Burrows-Wheeler Aligner (BWA) software. The resulting alignment files were sorted, filtered, and locally realigned to reduce false-positive variant calls. Variants of potential clinical significance were confirmed by Sanger sequencing.

### Genetic sequencing results

**Figure 1:**
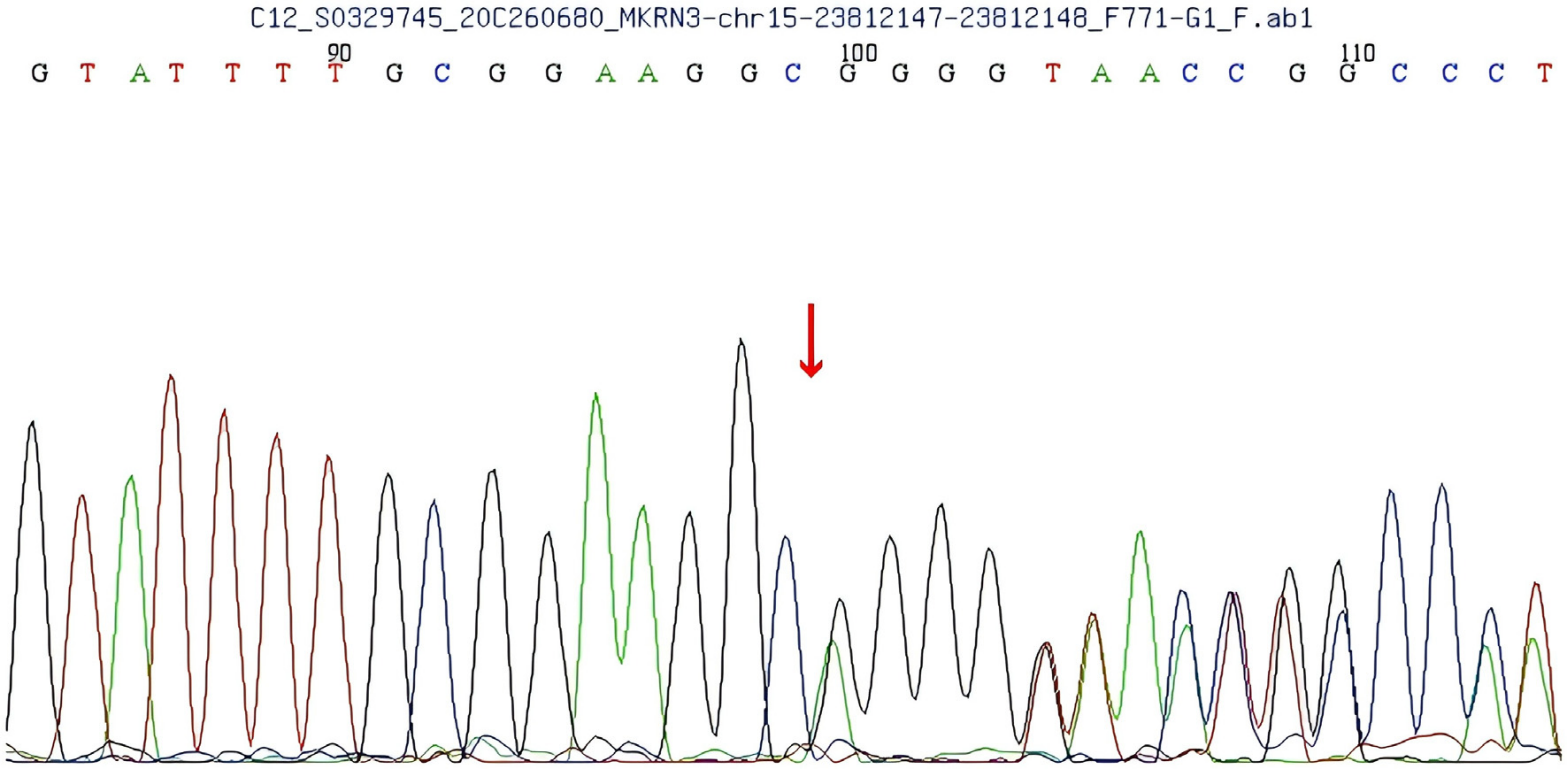
Sequencing results of the patient show a c.1219delA mutation in exon 1 of the makorin RING finger protein 3 (MKRN3) gene, leading to an Argine 407 to glycine (R407G) mutation.

**Figure 2:**
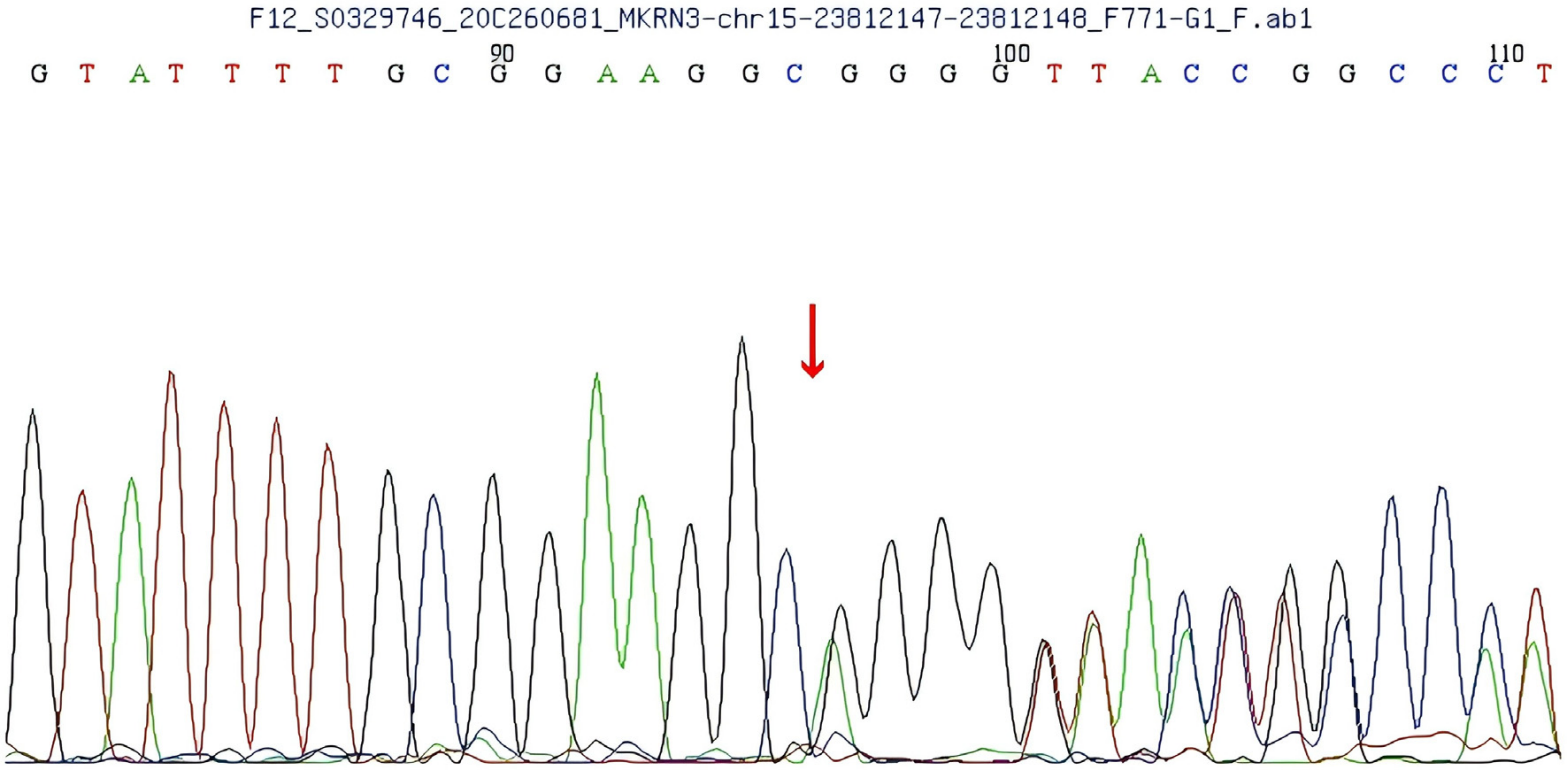
Gene sequencing of the patient’s father shows a single nucleotide deletion (1219delA) in exon 1 of the makorin RING finger protein 3 (*MKRN3*) gene, resulting in an Argine 407 to glycine (R407G) mutation.

**Figure 3:**
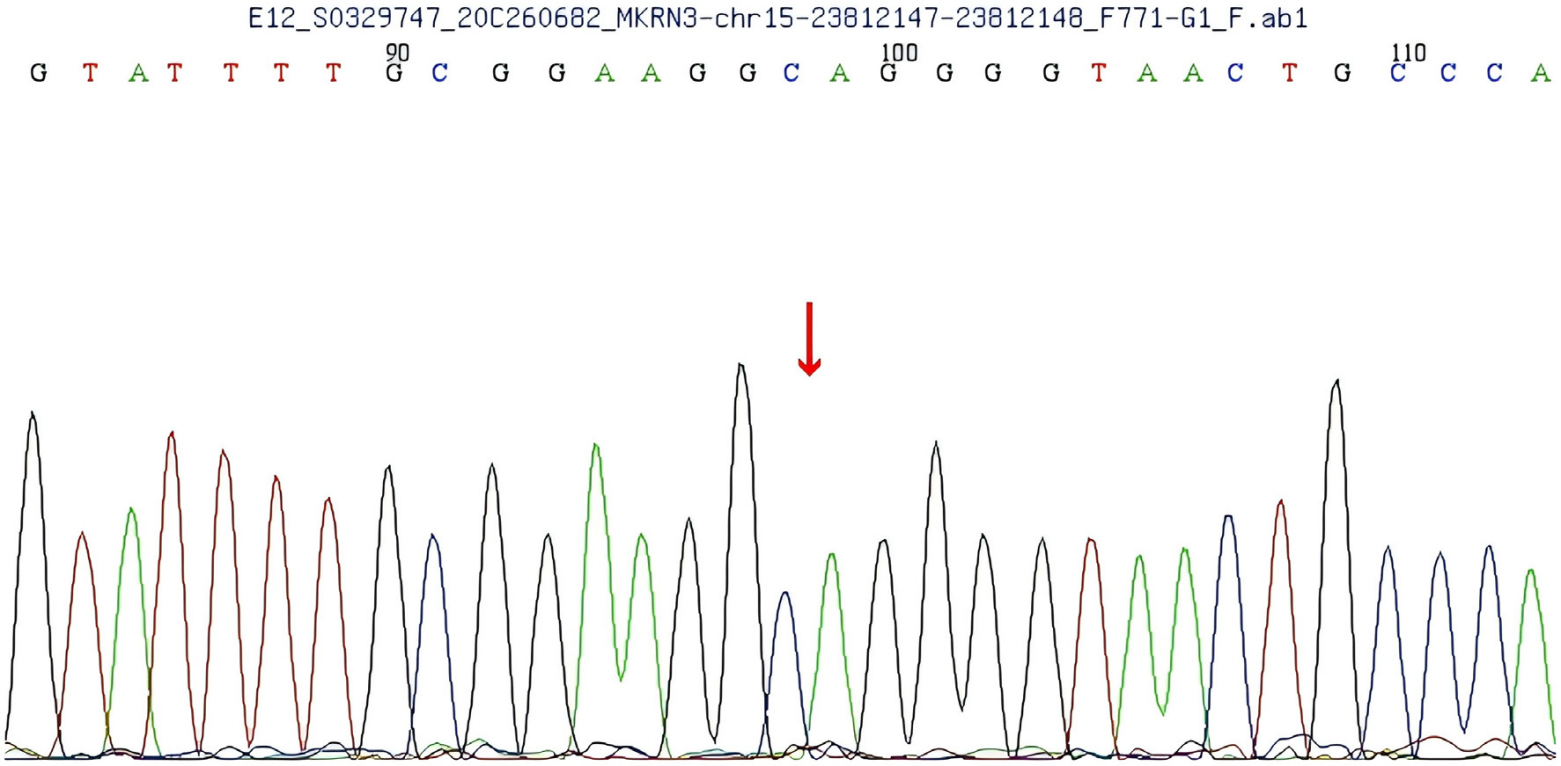
Gene sequencing of the patient’s mother shows no mutation in exon 1 of the makorin RING finger protein 3 (*MKRN3*) gene.

A heterozygous frameshift mutation (c.1219delA) was identified in exon 1 of the *MKRN3* gene (the reference transcript: NM_005664; exon1) in the proband (**Figure 1**). This single nucleotide deletion causes a frameshift that leads to the substitution of arginine (R) at codon 407 with glycine (G), ultimately resulting in premature termination of protein translation 73 amino acids downstream (p.R407Gfs*75). Familial segregation analysis confirmed that the proband’s father is a heterozygous carrier of the mutation, while the mother carries the wild-type (WT) allele at this locus (**Figures 2**, **3**).

This variant is absent from population databases and has not been previously reported in the Human Gene Mutation Database (HGMD). Additionally, ClinVar does not provide pathogenicity assessments for this variant. Based on the American College of Medical Genetics and Genomics (ACMG) guidelines, the variant is classified as likely pathogenic.

## Discussion

### Uniqueness of the case and discovery of a novel MKRN3 mutation

In this study, we report a case of a 4-year-9-month-old Chinese girl diagnosed with ICPP caused by a novel heterozygous frameshift mutation in the *MKRN3* gene (c.1219delA). This mutation leads to premature termination of the protein, resulting in a loss-of-function protein. The discovery of this mutation expands the known pathogenic mutation spectrum of the *MKRN3* gene, particularly in the Chinese population where data on *MKRN3* mutations are relatively scarce. This case provides a foundation for further exploration of the genetic characteristics and pathogenic mechanisms of MKRN3 in the Chinese population.

### Further insights into the pathogenesis of CPP

**Figure 4:**
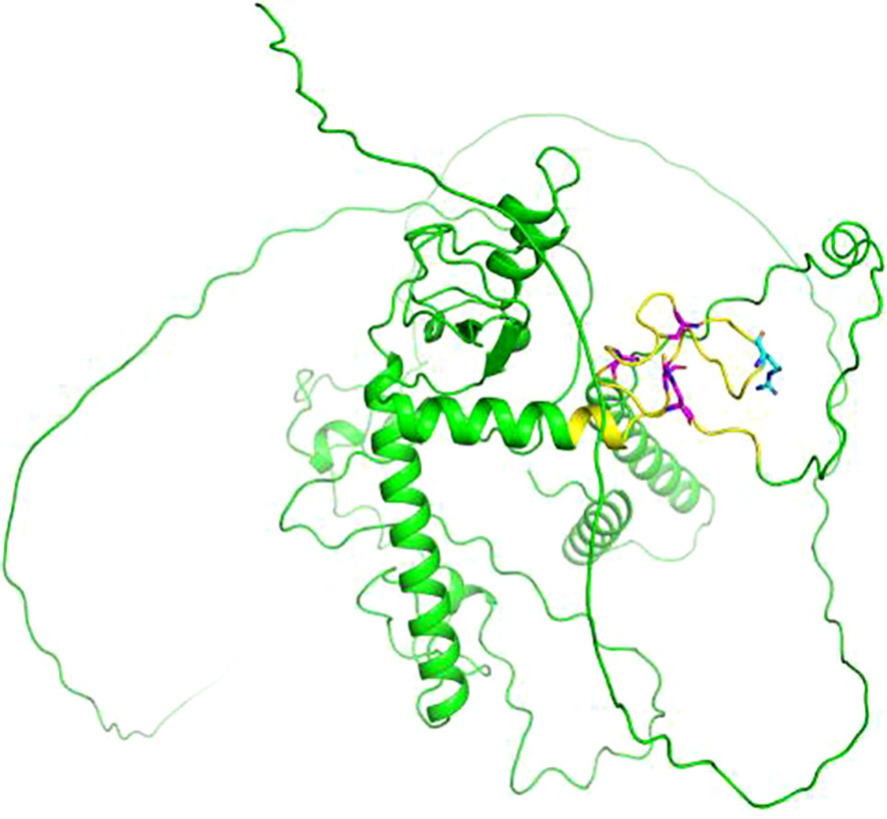
Cartoon representation of the three-dimensional (3D) structure of makorin RING finger protein 3 (MKRN3) predicted by AlphaFold (AF-Q13064-F1). The point mutation at position 407 is highlighted in cyan, and the C3H domain is highlighted in yellow. The cysteine (C) and histidine (H) residues of the C3H domain are highlighted in magenta. The image was generated using PyMOL (Version 2.5.5).

Previous studies have shown that the *MKRN3* gene plays an inhibitory role in suppressing GnRH secretion before puberty (7). In 2013, Abreu et al. pioneered the identification of pathogenic mutations in the *MKRN3* gene as a causative gene for CPP through WES analysis of 40 affected individuals from 15 families (8). They specifically identified four distinct *MKRN3* variants in 12 CPP patients across 5 families, including one missense and three frameshift mutations (8). Subsequent research across diverse populations has further expanded the repertoire of pathogenic *MKRN3* variants to a total of 54, with these mutations primarily classified as frameshift, missense, or nonsense (9–12) (**Table 2**). The novel mutation identified in this study is not located in the last base pairs, the mutated mRNA probable undergoes a nonsense mediated decay (NMD), and we consider that this novel discovered mutation would be a loss-of-function mutation. Although no experimental structural data is available for MKRN3 protein, the three-dimensional (3D) structure predicted by AlphaFold suggests that the R407G mutation occurs in the c-terminal C3H domain (**Figure 4**). This variant may retain E3 ubiquitin ligase activity but likely lacks RNA-binding capability due to the disruption of the C3H motif.

**Table 2:** Confirmed makorin RING finger protein 3 (*MKRN3*) Mutations.

Mutation site	Protein change	Type of mutation
c.683-684insA	–	reading frameshift
c.482insC	–	reading frameshift
c.802-803delAT	–	reading frameshift
c.482delC	–	reading frameshift
c.1053-1056delACAG	–	reading frameshift
c.477-485del	–	reading frameshift
c.475-476insC	–	reading frameshift
c.482-483insC	–	reading frameshift
c.675-676insA	–	reading frameshift
c.766-767delA	–	reading frameshift
c.637delC	–	reading frameshift
c.1171­1172insA	–	reading frameshift
c.441­441deJG	–	reading frameshift
c.630­650delinsGCTGGGC c.555­556delCA c.860delG	- - -	reading frameshift reading frameshift reading frameshift
c.1095G>T	p.Arg365Ser	missense mutation
c.1018T>G	p.Cys340Gly	missense mutation
c.1249T>A	p.Phe417Ile	missense mutation
c.1259T>G	p.His420Gln	missense mutation
c.1188C>A	p.Ser396Arg	missense mutation
c.699G>C	p.Lys233Asn	missense mutation
c.677A>C	p.Gln226Pro	missense mutation
c.611T>C	p.Ile204Thr	missense mutation
c.298A>T	p.Ile100Phe	missense mutation
c.587G>T	p.Gly196Val	missense mutation
c.1034G>A	p.Arg345His	missense mutation
c.737A>G	p.Tyr246Cys	missense mutation
c.1118C>T	p.Pro373Leu	missense mutation
c.89C>T	p.Pro30Leu	missense mutation
c.982C>T	p.Arg328Cys	missense mutation
c.943A>G	p.Met315Val	missense mutation
c.934G>A	g.Gly312Asp	missense mutation
c.1071C>G	p.Ile357Met	missense mutation
c.1138G>A	p.Glu380Lys	missense mutation
c.1420T>A	p.Leu474Met	missense mutation
c.673C>G c.939C>G c.749G>A c.203G>A c.1430G>A c.1212C>G c.1235T>G c.983G>A c.1276A>G c.350A>G c.376A>G c.1033C>T c.1091G>T c.1381A>T	p.Leu225Val p.Ile313Met p.Gly250Glu p.Arg68Phe p.Arg477Gln p.Ser368Cys P.Phe412Cys p.Arg328His p.Lys359Arg p.Tyr117Cys p.Met126Val p.Arg345Cys p.Cys364Phe p.Ile461Phe	missense mutation missense mutation missense mutation missense mutation missense mutation missense mutation missense mutation missense mutation missense mutation missense mutation missense mutation missense mutation missense mutation missense mutation
c.841C>T c.1087C>T c.891A>T c.331G>T	p.Gln281* p.Gln363* p.Glu298* p.Gln111*	nonsense mutation nonsense mutation nonsense mutation nonsense mutation

The exact role of *MKRN3* in regulating the central neuroendocrine system and how its mutations lead to CPP is still unclear. Studies in animals show that *MKRN3* can inhibit the secretion of gonadotropin-releasing hormone 1 (GnRH1). In mice, *MKRN3* mRNA levels peak around postnatal days 10-12 and then drop sharply by day 15, aligning with the start of puberty. Under normal conditions, *MKRN3* ubiquitinates methyl-CpG binding domain protein 3 (MBD3), disrupting its binding to the GnRH1 promoter. This prevents the recruitment of TET2, leading to increased promoter methylation and suppression of GnRH1 transcription. In humans, serum MKRN3 levels were found to exhibit substantial inter-individual variability but consistently decline prior to the onset of puberty, showing a negative correlation with gonadotropin secretion. This observation suggests that MKRN3 may exert an inhibitory effect on hypothalamic GnRH secretion during childhood (13). Decreased MKRN3 levels during puberty could lead to the release from suppression of the HPG axis, thereby triggering pubertal development (14). Moreover, some children with CPP exhibit markedly reduced or undetectable serum MKRN3 levels, potentially linked to *MKRN3* gene mutations (14). It is thus hypothesized that LOF mutations in *MKRN3* could compromise its inhibitory function over the HPG axis, leading to pulsatile GnRH release, premature activation of the axis, and consequently, precocious puberty. Further investigations into the relationship between MKRN3 and sex hormones have revealed an inverse correlation between MKRN3 levels and peripheral blood LH, FSH, and estradiol (E2) levels, but not with anti-Müllerian hormone (AMH) levels (15). These findings reinforce the concept that MKRN3 acts as a negative regulator of gonadotropins and E2, suppressing hypothalamic GnRH secretion during childhood and declining prior to pubertal onset. Elucidating the expression patterns and functional roles of MKRN3 protein is crucial for understanding the regulatory mechanisms underlying human pubertal initiation.

### Clinical management implications of this case

The mutation in this patient was inherited from her asymptomatic father, consistent with the imprinting inheritance pattern of the *MKRN3* gene. Since *MKRN3* is a paternally expressed imprinted gene, mutations are typically passed from asymptomatic fathers to their offspring. Understanding the family’s genetic background, especially the developmental history of the father, is crucial for accurate diagnosis and family genetic counseling. This study highlights the importance of comprehensive genetic analysis in early-onset ICPP cases to accurately identify pathogenic mutations.

Identifying the genetic cause of CPP early is crucial for personalized treatment. The mutation in this patient has not been reported in public databases, indicating that it is a novel pathogenic variant. For cases like this, clinicians should consider early genetic testing to formulate personalized management strategies. Moreover, as CPP caused by *MKRN3* mutations tends to have an earlier onset, close monitoring and follow-up are necessary, especially for asymptomatic individuals from the same family, particularly paternal family members, to ensure timely intervention.

### Therapeutic and prognostic outlook

Currently, GnRH analogs are the standard treatment for CPP. These analogs exert their therapeutic effect primarily at the anterior pituitary, where they competitively bind to GnRH receptors on gonadotrophs. While an initial transient increase in LH, FSH, and sex hormones may be observed, continuous administration leads to pituitary desensitization, reducing gonadotropin secretion and subsequently suppressing sex hormone levels, thus effectively interrupting the prematurely activated HPG axis.

Importantly, extended GnRH analog therapy has been recommended for individuals with CPP caused by *MKRN3* gene mutations (16). While the therapeutic response to GnRH analogs in MKRN3-induced CPP is generally comparable to that observed in idiopathic CPP, the earlier average age of onset in MKRN3-related cases necessitates vigilant monitoring of even asymptomatic children from affected families. This proactive approach ensures the timely identification of CPP onset and allows for prompt intervention, optimizing treatment outcomes.

Although GnRH analogs remain the primary treatment for CPP, long-term efficacy and therapeutic strategies for CPP caused by *MKRN3* mutations may need further optimization. Since early intervention is critical in preventing premature epiphyseal closure and limiting adult height, this study supports the importance of early genetic screening and individualized treatment for such cases.

## Conclusions

By linking this case to the specific findings, this study not only adds to the body of evidence on the genetic basis of CPP but also provides new insights and directions for future clinical management and research. This study underscores the importance of *MKRN3* mutations in the Chinese population, but further functional studies are needed to elucidate the precise pathophysiological mechanisms. These studies will help improve our understanding of the genetic mechanisms underlying CPP and lead to more precise therapeutic approaches.

## Data Availability

The original contributions presented in the study are included in the article/supplementary material, further inquiries can be directed to the corresponding author/s.
